# Efficacy and Safety of Modified Banxia Xiexin Decoction (Pinellia Decoction for Draining the Heart) for Gastroesophageal Reflux Disease in Adults: A Systematic Review and Meta-Analysis

**DOI:** 10.1155/2017/9591319

**Published:** 2017-02-19

**Authors:** Yunkai Dai, Yunzhan Zhang, Danyan Li, Jintong Ye, Weijing Chen, Ling Hu

**Affiliations:** Institute of Gastroenterology, Guangzhou University of Chinese Medicine, Guangzhou, Guangdong, China

## Abstract

Modified Banxia Xiexin decoction (MBXD) is a classical Chinese herbal formula in treating gastroesophageal reflux disease (GERD) for long time, but the efficacy of it is still controversial. This study is to evaluate the efficacy and safety of MBXD for the treatment of GERD in adults. The search strategy was carried out for publications in seven electronic databases. RevMan software version 5.3 and the Cochrane Collaboration's risk of bias tool were performed for this review. Twelve RCTs were included for the analysis. The results of overall clinical efficacy and efficacy under gastroscope demonstrated that MBXD was superior to conventional western medicine. Meanwhile, the results of subgroup analysis showed clinical heterogeneity between the two groups. However, there was no statistically significant difference in acid regurgitation between the two groups. But in the improvement of heartburn and sternalgia, the results showed statistically significant differences for the comparison between two groups. In addition, the adverse reactions of the experiment groups were not different from those of the control groups. This systematic review indicates that MBXD may have potential effects on the treatment of patients with GERD. But because the evidence of methodological quality and sample sizes is weak, further standardized researches are required.

## 1. Introduction

Gastroesophageal reflux disease (GERD), which affects a substantial proportion of the world's population particularly in western countries, is defined as a gastroesophageal motility disorder that appears when the reflux of stomach contents causes troublesome gastroesophageal symptoms and/or complications [1]. Based on its clinical manifestation, GERD is subclassified into three types: nonerosive reflux disease (NERD), reflux esophagitis (RE), and Barrett esophagus (BE) [2].

According to epidemiological investigation [3], the prevalence of symptom-based GERD increased from 2.5–4.8% before 2005 to 5.2–8.5% from 2005 to 2010 in East Asia, and after 2005, the prevalence was 6.3–18.3% in Southeast and West Asia. Similarly, in East Asia, the prevalence of endoscopic reflux esophagitis increased from 3.4–5.0% to 4.3–15.7%. Thus, the incidence of GERD appears to be an increasing problem throughout Asia including China, causing substantial reductions in subjective wellbeing [4] and lower work productivity and involving substantial healthcare costs [5].

Proton pump inhibitors (PPIs) are currently the mainstay of treatment for GERD. To be better control of acid secretion, a substantial proportion of patients require twice-daily therapy with PPIs. In addition, decreasing transient lower esophageal sphincter relaxations (TLESRs) can reduce distal acid exposure and weakly acidic refluxate [6]. Despite the efficacy of these agents in healing and symptom relief, many Asian patients with GERD continue to experience symptoms [7]. Moreover, the long-term use of PPIs may cause some clinical risks, such as fracture [8–10], respiratory infection [11–13], spontaneous peritonitis [14], and clostridium difficile bacteria infection [15–17].

Due to chronicity and progressivity of GERD, many patients have turned their attentions to traditional Chinese medicine (TCM) [18, 19]. Modified Banxia Xiexin decoction (MBXD), an ancient formula in treating GERD [20], is modified by different Chinese herbal additions based on Banxia Xiexin decoction according to TCM syndrome differentiation. However, in the past decades, although numerous studies have compared MBXD with conventional western medicine in the treatment of GERD, the comparability of treatment protocols and evaluation methodologies among these studies remains to be proven, which greatly limits their clinical applicability [21]. Furthermore, the current state of evidence of MBXD for GERD has so far been unknown. Therefore, we conducted this systematic review to evaluate efficacy and safety of MBXD in the treatment of GERD.

## 2. Materials and Methods

### 2.1. Eligibility Criteria

The studies included in this review were randomized controlled trials (RCTs) in humans, without limitations on publication type. And all the included studies should present the efficacy of MBXD in comparison with conventional western medicine. Outcomes should contain at least one outcome, such as overall clinical efficacy, efficacy under gastroscope, or symptom scores. In addition, overall clinical efficacy was our primary outcome in this systematic review.

### 2.2. Patients

GERD is diagnosed on the basis of published diagnostic criteria [22]. All patients in the included studies had confirmed diagnoses of it. In addition, pregnant women, juveniles, and patients with malignant tumour or severe cardiovascular diseases were excluded.

### 2.3. Databases and Search Strategy

A literature search was comprehensively carried out for publications in the following 7 electronic databases from their inception through July 30, 2016: PubMed, Embase, Springer Link, CNKI (China National Knowledge Infrastructure), VIP (Chinese Scientific Journals Database), Wan-fang database, and CBM (Chinese Biomedicine Database). In the article search, the following general wordings of search terms were used individually or in combination: “gastroesophageal reflux disease”, “reflux esophagitis”, “nonerosive gastroesophageal reflux disease”, “barrett's esophagus”, “Banxia Xiexin decoction”, “traditional Chinese medicine”, “herbal formula”, “herbs”, “clinical application”, “randomized controlled trials”, and “clinical trial”. No limit for publication was placed on language. Manual searches of relevant literatures supplemented the electronic searches.

### 2.4. Endpoint Indicators

Dichotomous data in this systematic review contained overall clinical efficacy and efficacy under gastroscope. Both of them were graded into 3 or 4 categories according to the appropriate guiding principles and guidelines [22–24]: (cure), markedly effective, effective, and ineffective.

### 2.5. Study Identification

Two investigators (Yunkai Dai and Yunzhan Zhang) independently extracted data from all included publications, including the first author, publication year, classification of GERD, sample size, age, course of disease, duration, intervention, outcome measures, randomization, double blinding, withdrawal or dropout, allocation concealment, follow-up, and side effects. Data were extracted as intention-to-treat (ITT) analyses, in which dropouts were assumed to be treatment failures. One researcher (Yunkai Dai) extracted the initial data; the other (Yunzhan Zhang) subsequently reexamined each study and verified the results. Disagreements were resolved by discussion with another researcher (Danyan Li).

### 2.6. Quality Assessment

Evaluation of methodological quality in the included studies was performed independently by two reviewers (DYL and JTY), which used the Cochrane Collaboration's risk of bias tool [25], supplemented by Jadad score [26]. We could judge whether all the included literatures contained selection bias, performance bias, detection bias, attrition bias, reporting bias, and other bias from randomization, double blinding, and withdrawal or dropout. Literature with a Jadad score above 3 was regarded as a superior quality article; otherwise, it was viewed as a poor one. However, the final results of literature quality including the risk of bias evaluation were illustrated by the Cochrane tool.

### 2.7. Data Synthesis and Analysis

This systematic review used Review Manager 5.3 software to pool effect sizes. Summary odds ratio (OR) or risk ratio (RR) and 95% confidence intervals (CI) were calculated for overall clinical efficacy, efficacy under gastroscope, and recurrence rate. Standardized mean difference (SMD) or mean difference (MD) and 95% CI were reported for symptom scores. Heterogeneity was evaluated statistically using the *χ*^2^ test and inconsistency index statistic (*I*^2^) [27]. If substantial heterogeneity existed (*I*^2^ > 50% or *P* < 0.05), a random effect model was applied. If there was no observed heterogeneity, fixed effect models were chosen [28]. A sensitivity analysis was done to explore potential sources of heterogeneity. Publication bias was evaluated using visual inspection with the aid of a funnel plot.

## 3. Results

### 3.1. Description of Studies

**Figure 1:**
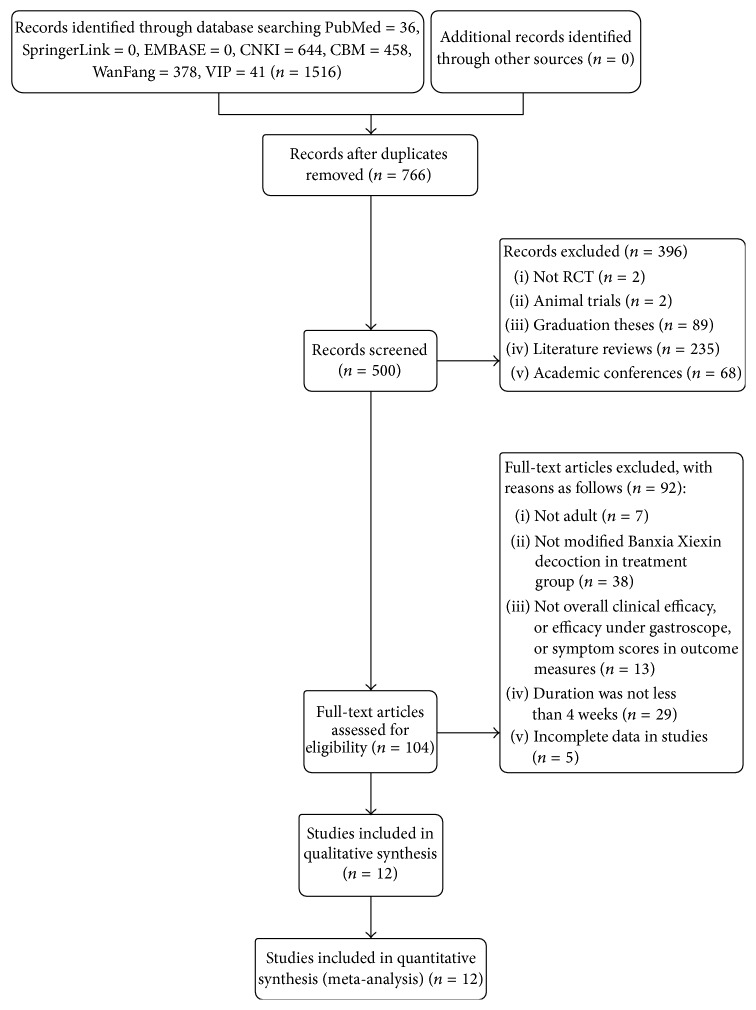
Flowchart of the process for literature retrieval.

A total of 1516 records were obtained based on the search strategy. After further screening, 12 RCTs (*N* = 1210) satisfied the inclusion criteria and were included in this meta-analysis [29–40]. The flowchart of search process and study selection was shown in Figure 1. In addition, 12 studies were published in Chinese. Sample sizes ranged from 60 [34] to 150 [39]. The ages of patients are from 18 to 72 years. The courses of disease were between 2 days and 30 years apart from 2 studies [35, 37] without mention. The therapeutic sessions ranged from 4 weeks [33, 36, 38] to 8 months [39]. In addition, as for classification of GERD, NERD was reported by 1 study [29], RE was reported by 7 studies [31, 35–40], and the remaining four studies [30, 32–34] did not mention the classification of GERD. The characteristics of the included studies were presented in Table 1. The constituents of herbal formulae were listed in Table 2.

### 3.2. Risk of Bias Assessment

**Figure 2:**
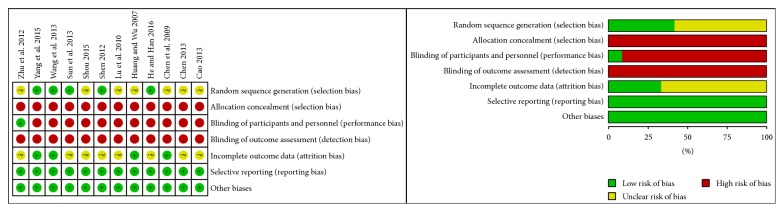
Risk of bias summary and graph.

All of the 12 included RCTs described no significant differences at baseline between experiment groups and treatment groups. However, only 5 studies [29, 31, 32, 34, 37] reported a randomization technique using random number table, while the other 7 [30, 33, 35, 36, 38–40] did not report the specific randomization technique. Moreover, none of the 12 trials described double blinding and allocation concealment. Although only 1 trial [36] mentioned a single-blind design, and the specific implementation of this design was not reported. In addition, dropouts were described in 2 trials [29, 36], but neither of them performed ITT analysis. In general, owing to the relative lacking of specific information (Figure 2), the validity of this meta-analysis was regarded as high risk. A description of the evaluation of methodological quality of the 12 trials can be found in Table 3.

### 3.3. Primary Outcome: Comparison of Overall Clinical Efficacy

**Figure 3:**
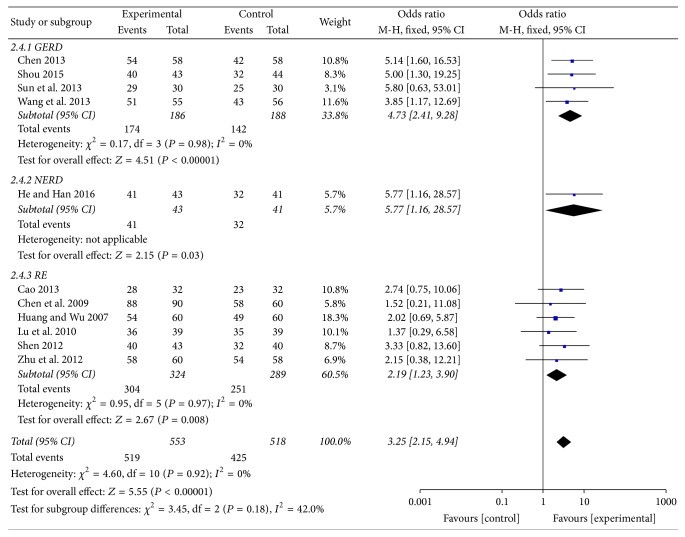
Forest plot of overall clinical efficacy (fixed effect model).

**Figure 4:**
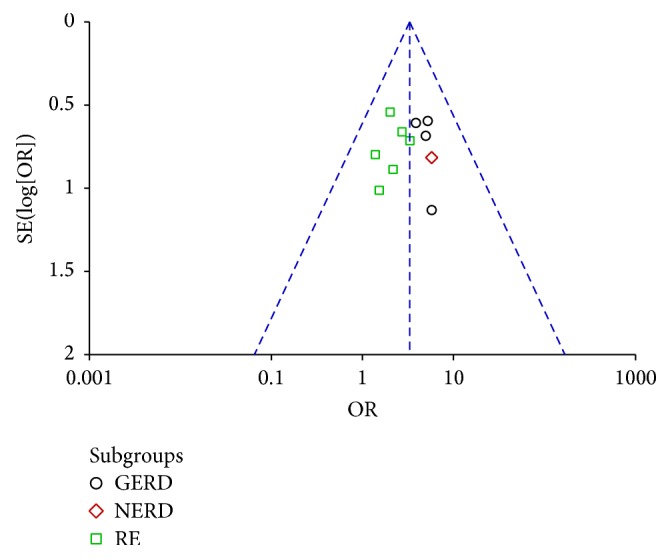
Funnel plot of overall clinical efficacy.

Among the included studies, eleven including 1071 patients (553 in the experiment groups versus 518 in the control groups) evaluated overall clinical efficacy [29, 30, 32–40]. On subgroup meta-analysis, 4 trials [30, 32–34] reported GERD, 1 trial [29] reported NERD, and 6 trials [31, 35–40] reported RE, and all of them showed statistically significant differences between MBXD and conventional western medicine (OR 3.25; 95% CI: 2.15 to 4.94; *P* < 0.00001). In addition, because of good homogeneity (*χ*^2^ = 4.60,  *P* = 0.92, *I*^2^ = 0%), a fixed effect model was adopted to estimate pooled effect size for the analysis (Figure 3). The symmetrical funnel plot showed no potential publication bias in Figure 4.

#### 3.3.1. Subgroup Analysis

**Figure 5:**
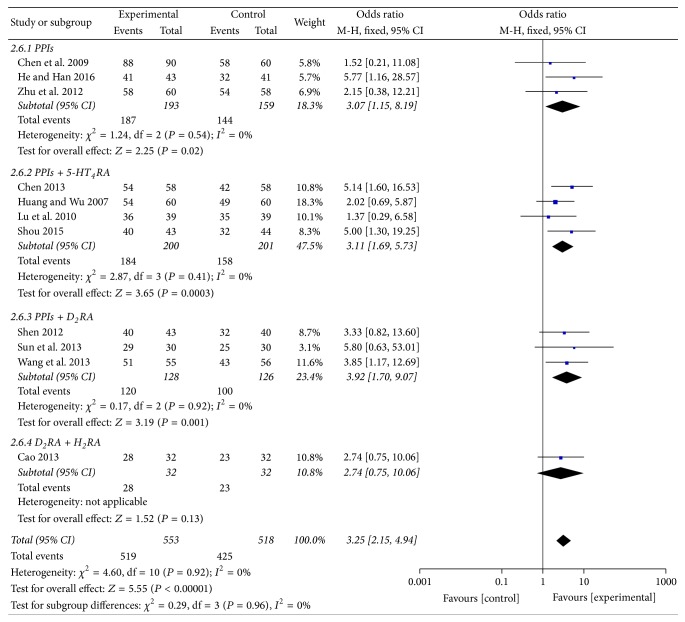
Forest plot of subgroup analysis (fixed effect model).

**Figure 6:**
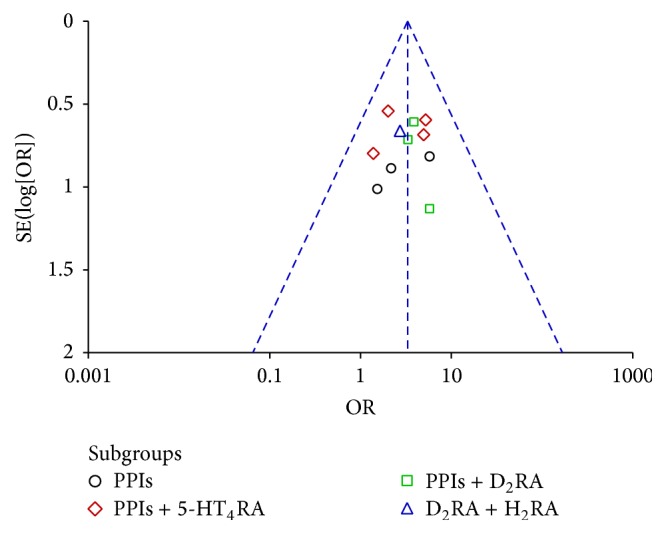
Funnel plot of subgroup analysis.

Because of variability in evaluating point of the efficacy, we conducted subgroup analysis among studies using different conventional western medicines of PPIs, PPIs + 5-HT_4_ receptor agonists (5-HT_4_RA), PPIs + D_2_ receptor antagonists (D_2_RA), and D_2_RA + H_2_ receptor antagonists (H_2_RA). In the included studies, PPIs contained omeprazole, lansoprazole, pantoprazole, and rabeprazole; 5-HT_4_RA contained mosapride and cisapride; D_2_RA contained domperidone; H_2_RA contained ranitidine. Compared with the control groups, the results of subgroup analysis showed clinical heterogeneity between MBXD and PPIs (OR 3.07; 95% CI 1.15, 8.19; *P* = 0.02) in three trials [29, 36, 39], between MBXD and PPIs + 5-HT_4_RA (OR 3.11; 95% CI 1.69, 5.73; *P* = 0.0003) in four trials [30, 33, 38, 40], between MBXD and PPIs + D_2_RA (OR 3.92; 95% CI 1.70, 9.07; *P* = 0.001) in three trials [32, 34, 37], between MBXD and D_2_RA + H_2_RA (OR 2.74; 95% CI 0.75, 10.06; *P* = 0.13) in one trial [35], and an overall clinical efficacy (OR 3.25; 95% CI 2.15, 4.94; *P* < 0.00001) in Figure 5. A funnel plot analysis of the 11 trials [29, 30, 32–40] suggested possible publication bias and inclusion of low quality studies because of a significant asymmetry as shown in Figure 6.

#### 3.3.2. Sensitivity Analysis

Because of good homogeneity in primary outcome (*I*^2^ = 0% for overall clinical efficacy), we did not conduct a sensitivity analysis for overall clinical efficacy.

### 3.4. Secondary Outcomes

#### 3.4.1. Comparison of Efficacy under Gastroscope

**Figure 7:**
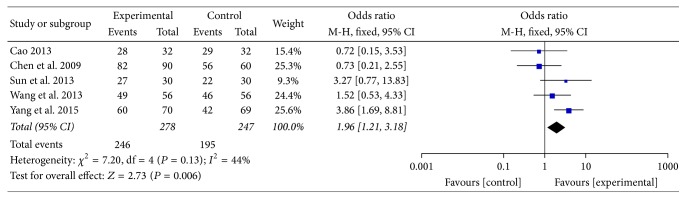
Forest plot of efficacy under gastroscope (fixed effect model).

**Figure 8:**
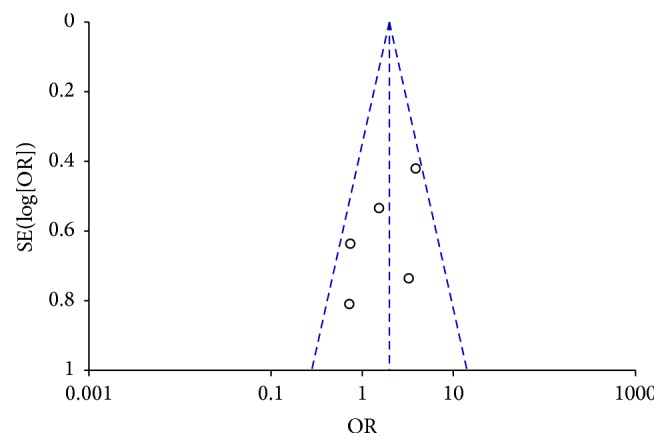
Funnel plot of efficacy under gastroscope.

Five of twelve studies reported the efficacy under gastroscope [31, 32, 34, 35, 39]: of 525 patients, 278 were assigned to the groups of MBXD, whereas 247 were assigned to the groups of conventional western medicine. A model of fixed effect was performed to pool estimates because the meta-analysis indicated that *I*^2^ = 44%. The treatment groups showed moderate heterogeneity in efficacy under gastroscope compared to the control groups (OR 1.96; 95% CI: 1.21 to 3.18; *P* = 0.006) (Figure 7). The asymmetrical funnel plot in Figure 8 presented potential publication bias.

### 3.5. Improvement of Symptom Scores

Of all the included trials, eight reported the improvement of symptom scores [30, 31, 33–36, 38, 39]. Although eight studies evaluated the improvement of acid regurgitation [30, 31, 33–36, 38, 39], three were excluded because of different scoring criteria compared with the remaining five studies [30, 33, 34]. Moreover, six studies evaluated the heartburn improvement [31, 34–36, 38, 39], but due to differences in scoring criteria, one was excluded [34]. In addition, six studies described the improvement of sternalgia [30, 31, 34–36, 38], but four were excluded because of being different from the remaining two studies in scoring criteria [30, 34–36]. As for other improvements of symptom scores, these were analyzed qualitatively because only one study respectively described them. However, although the improvements of acid regurgitation, heartburn, and sternalgia were scored by the appropriate guiding principle [23], the scores of them were classified as 0~3′, 0~6′, or 0~9′. Therefore, subgroup analysis was conducted for each symptom score.

#### 3.5.1. Acid Regurgitation

**Figure 9:**
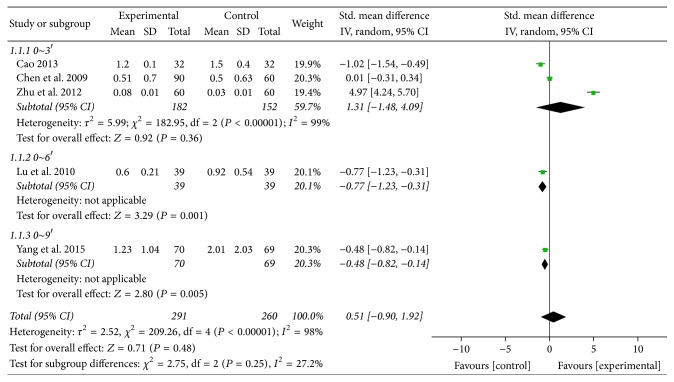
Forest plot of acid regurgitation (random effect model).

For the reduction of the scores of acid regurgitation, five trials [31, 35, 36, 38, 39] adopted random effect models to estimate pooled effect size for significant heterogeneity (*χ*^2^ = 209.26, *P* < 0.00001, *I*^2^ = 98%) (Figure 9). Furthermore, we can conclude from Figure 9 that acid regurgitation improvement had no statistically significant difference for the comparison between experiment groups and control groups (SMD 0.51; 95% CI: −0.90 to 1.92; *P* = 0.48).

#### 3.5.2. Heartburn

**Figure 10:**
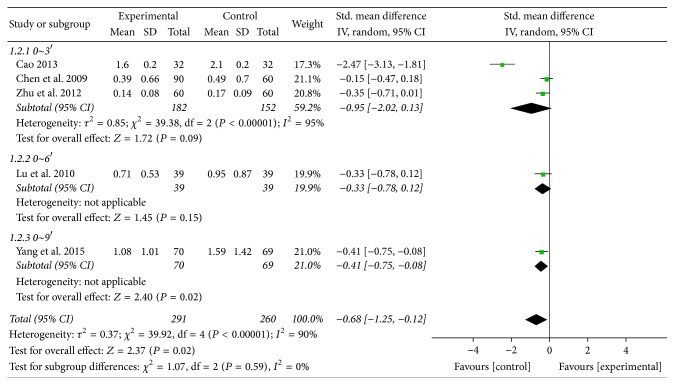
Forest plot of heartburn (random effect model).

The five studies as mentioned above also reported heartburn [31, 35, 36, 38, 39]. But because of significant heterogeneity in heartburn score (*χ*^2^ = 39.92, *P* < 0.00001, *I*^2^ = 90%), a random effect model was performed. Meanwhile, the reduction of heartburn score showed statistically significant difference between treatment groups and control groups (SMD −0.68; 95% CI: −1.25 to −0.12; *P* = 0.02) (Figure 10).

#### 3.5.3. Sternalgia

**Figure 11:**
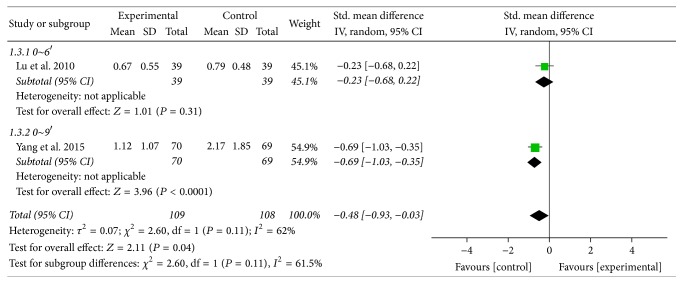
Forest plot of sternalgia (random effect model).

For the improvement of sternalgia, two trials used a model of random effect for the existence of significant heterogeneity (*χ*^2^ = 2.60, *P* = 0.11, *I*^2^ = 62%) [31, 38]. Moreover, the forest plot of sternalgia presented statistically significant difference between MBXD and conventional western medicine (SMD −0.48; 95% CI: −0.93 to −0.03; *P* = 0.04) (Figure 11).

#### 3.5.4. Recurrence Rate

**Figure 12:**
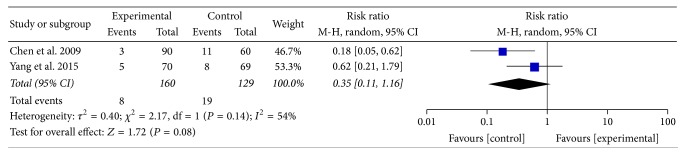
Forest plot of recurrence rate (random effect model).

In the included studies, although four reported the follow-up after treatment (Yang et al. for 6 months, Wang et al. for 1 week, Chen et al. for 12 weeks, and Huang and Wu for 3 months) [31, 32, 39, 40], only two trials mentioned recurrence rate during the period of follow-up [31, 39]. Furthermore, the forest plot of recurrence rate using random effect models showed no statistically significant difference in Figure 12 (RR 0.35; 95% CI: 0.11 to 1.16; *P* = 0.08).

#### 3.5.5. Adverse Events

Of all the included RCTs, three reported adverse reactions during the treatment period [36, 38, 39]. However, one trial mentioned no adverse events [40]; the other two mentioned the number of people in adverse effects (Zhu et al. for 8 cases and Chen et al. for 14 cases) [36, 39]. Furthermore, the Zhu et al. study reported that 3 cases suffered from diarrhea and 5 cases suffered from abdominal distention. The Chen et al. study reported that 4 cases developed nausea, 7 cases developed headache, 2 cases developed abdominal pain, and 1 case developed soreness of waist. Although these side effects occurred in the period of treatment, they did not have impact on the experimental process.

## 4. Discussion

This meta-analysis included 12 studies with 1210 total participants comparing MBXD with conventional western medicine for the treatment of GERD. As for the overall clinical efficacy and efficacy under gastroscope, our analysis revealed that experiment groups showed better efficacy than control groups. Meanwhile, the results of subgroup analysis showed clinical heterogeneity between MBXD and conventional western medicine. However, as for the improvements of acid regurgitation, heartburn, and sternalgia, the result of meta-analysis in acid regurgitation had a similar efficacy when compared with the control groups. But the results of meta-analyses in heartburn and sternalgia showed better improvement than conventional western medicine. In addition, both recurrence rate and adverse events had no statistically significant difference between treatment groups and control groups. Moreover, weaknesses were identified in most trials using the Cochrane Collaboration's risk of bias tool, while the quality level of Jadad score evaluation indicated “poor.” In a word, although MBXD had a positive therapeutic effect on overall clinical efficacy and efficacy under gastroscope, because of the high risk of bias of the included studies, the significant differences observed in this systematic review may be inaccurate. Therefore, further research must be required to acquire specific evidence for efficacy and safety of MBXD in treating GERD.

The pathogenesis of GERD remains inadequately explained. Previous studies have demonstrated that numerous potential mechanisms are involved in the development of GERD, including histologic changes of esophageal inflammation [41], antireflux barrier dysfunction [42], obesity [43], psychological factors [44, 45], hiatal hernia [46], and transient lower esophageal sphincter relaxation (TLESR) [47]. However, our studies, in modern pharmacological field, are consistent with the evidence for the effectiveness of MBXD for GERD. Experimental data have verified that MBXD can relieve esophageal mucosa injury and reduce the expression of intercellular adhesion molecule-1 and L-selectin in rats with RE [48]. Other data suggest that pungent dispersion bitter purgation (Xinkai Kujiang) method can present favorable treatment effect on RE model rats and the therapeutic effect may be more obvious along with the treatment course that went by, possibly by achieving through good repair effect on damaged mucosa, increasing the pressure of esophageal sphincter, and inhibiting gastric acid [49]. In addition, a few studies have shown that MBXD can exert its preventive and protective effect on esophageal mucosa by downregulating mRNA expression for calponin and caldesmon, increasing the intracellular free calcium, lowering gastric acidity with modulation of calcitonin- gene-related peptide synthesis in rats with RE [50, 51]. In a word, MBXD may be a multitargeting management in treating GERD. To better understand the herbal formulae mechanism, further studies in vitro and in vivo should be conducted.

There was significant heterogeneity for secondary outcomes. We checked all of the included studies carefully and found that there was difference of scoring criteria for symptom scores among them. Furthermore, the scores of acid regurgitation, heartburn, and sternalgia were categorized into three different levels (0~3′, 0~6′, or 0~9′), which may be the main origin of the heterogeneity. In addition, in the included trials, five reported the improvement of acid regurgitation and heartburn [31, 35, 36, 38, 39], and two reported sternalgia improvement [31, 38]. The quantity of the literatures in this systematic review was too small to yield reliable results, which may contribute to the heterogeneity.

Most evaluations of Chinese medicinal herbs have focused on the efficacy of diseases. And treatment based on syndrome differentiation is a characteristic of TCM. However, the information for TCM syndrome classification was taken into consideration only in five trials [29, 32, 33, 37, 38] and these trials presented variations in TCM syndrome classification. Furthermore, of the included twelve trials, although all the Chinese herbal formulae in treatment groups were based on Banxia Xiexin decoction, MBXD contained different additional herb(s). Moreover, the doses, frequencies, and methods of administration were different among these trials. In addition, discrepancies in the herbal medicines themselves including source and preparation were existent. In sum, all of them mentioned above could be a matter of heterogeneity among the evaluated studies.

Several limitations of this systematic review were as follows: First, single center, small sample size, and low methodological quality resulted in poor quality of the included RCTs. Moreover, all of the participants in the included trials were Chinese. This geographically limited distribution and poor quality of studies were hard to apply in future large-scale trials. As for the evaluation of publication bias, the power of this systematic review was modest because of the small number of studies, resulting in the possible existence of publication bias for the analysis. Second, only four trials reported the follow-up visits and the follow-up periods were between 1 week and 6 months [31, 32, 39, 40]. In addition, the treatment courses in the twelve studies ranged from 4 weeks to 8 months. Both the follow-up periods and treatment courses were not long enough to assess the long-term efficacy and safety of MBXD for GERD. Third, dropouts from the RCTs were reported only in two trials [29, 36], and the missing data were not evaluated by ITT analysis, which produced deviation in assessment of the efficacy of interventions. Fourth, only two trials reported recurrence rate [31, 39] and three trials reported side effects [36, 38, 39]. The minority of literatures reported recurrence rate and side effects, which potentially caused unreliable results and inability to truly reflect general trends. Fifth, discrepancies in interventions among control groups existed. Therefore, potential harm for all medical drugs should be taken into consideration.

## 5. Conclusion

Evidence from this systematic review shows that MBXD has a positive efficacy in the treatment of GERD. However, because of limitations of methodological quality and small number of the included studies, recommendations of specific MBXD for GERD cannot be made at present, and these results should be interpreted cautiously. Therefore, further standardized researches with multicenter, large-scale, and rigorous design are needed.

## Figures and Tables

**Table 1:** Characteristics of the studies included in the meta-analysis.

Study ID (first author, year)	Classification of GERD	Type of syndrome	Sample size		Age (years)	Course of disease	Duration	Intervention		Outcome measures
			EG (M/F)	CG (M/F)				EG	CG	
He and Han 2016 [29]	NERD	Stagnated heat in liver and stomach syndrome	45 (26/19)	45 (29/16)	21–68	2 days–102 days	8 weeks	Modified Banxia Xiexin decoction, 100 mL, b.i.d	PPIs	①④⑤
Shou 2015 [30]	N/A	N/A	43 (24/19)	43 (23/20)	E: 51.7 ± 12.9 C: 52.6 ± 12.9	1 year–10 years	60 d	Banxia Xiexin decoction, 100 mL, t.i.d	PPIs + 5-HT_4_RA	①⑥
Yang et al. 2015 [31]	RE	N/A	70 (38/32)	69 (35/34)	E: 41.89 ± 5.67 C: 40.31 ± 6.98	E: 4.56 ± 1.23 C: 4.09 ± 1.68	3 months	Modified Banxia Xiexin decoction plus Sini Powder, b.i.d	PPIs + D_2_RA	②③⑥
Wang et al. 2013 [32]	N/A	Cold and heat mixed type	56 (30/26)	56 (32/24)	22–64	1 year–12 years	8 weeks	Modified Banxia Xiexin decoction, b.i.d + point injection (vitamin B6), q.o.d	PPIs + D_2_RA	①②⑦
Chen 2013 [33]	N/A	Stagnation of liver and stomach Qi, stomachache due to cold, deficiency of stomach, yin, hyperactivity of stomach, heat, syndrome of retention of food in stomach	E:C 58/58 M:F 70/46		21–68	0.5 year–13 years	4 weeks	Modified Banxia Xiexin decoction, b.i.d	PPIs + 5-HT_4_RA	①⑥
Sun et al. 2013 [34]	N/A	N/A	30 (17/13)	30 (14/16)	21–61	1 year–6 years	30 d	Modified Banxia Xiexin decoction	PPIs + D_2_RA	①②⑥⑧
Cao 2013 [35]	RE	N/A	32 (26/6)	32 (22/10)	Mentioned	N/A	8 weeks	Modified Banxia Xiexin decoction, 1 dose/d	D_2_RA + H_2_RA	①②⑥
Zhu et al. 2012 [36]	RE	N/A	60 (32/28)	60 (29/31)	19–72	3 months–12 years	4 weeks	Banxia Xiexin decoction plus Xuanfu Daizhe decoction, 250 mL, b.i.d	PPIs	①⑥
Shen 2012 [37]	RE	Cold and heat mixed type	43 (27/16)	40 (26/14)	26–70	N/A	56 days	Modified Banxia Xiexin decoction, 100 mL, b.i.d	PPIs + D_2_RA	①⑨
Lu et al. 2010 [38]	RE	Stomach Qi rising	39 (20/19)	39 (19/20)	20–65	1–6 years	4 weeks	Modified Banxia Xiexin decoction, b.i.d	PPIs + 5-HT_4_RA	①②⑥
Chen et al. 2009 [39]	RE	N/A	90 (56/34)	60 (39/21)	18–69	2–10 weeks	8 months	Modified Banxia Xiexin decoction, 150 mL, b.i.d	PPIs	①②③⑥⑨
Huang and Wu 2007 [40]	RE	N/A	60 (35/25)	60 (38/22)	18–61	2 months–30 years	8 weeks	Modified Banxia Xiexin decoction, b.i.d	PPIs + 5-HT_4_RA	①

①: overall clinical efficacy; ②: efficacy under gastroscope; ③: recurrence rate; ④: RDQ, SAS, and SDS grading; ⑤: SF-36 dimensions of grading; ⑥: symptom integrals; ⑦: plasma GAS level; ⑧: pathological effect; ⑨: RE classification in gastroscopy; GERD: gastroesophageal reflux disease; NERD: nonerosive reflux disease; RE: reflux esophagitis; M: male; F: female; EG: experiment group; CG: control group; N/A: not applicable; RDQ: reflux disease diagnostic questionnaire; SAS: self-rating anxiety scale; SDS: self-rating depression scale; PPIs: proton pump inhibitors; 5-HT_4_RA: 5-HT_4_ receptor agonists; D_2_RA: D_2_ receptor antagonists; H_2_RA: H_2_ receptor antagonists.

**Table 2:** The ingredients of each formula.

Author	Ingredients of each formula			
He and Han 2016 [29]	*Scutellaria baicalensis *Georgi	*Fritillaria thunbergii *Miq.	*Taraxacum mongolicum *Hand.-Mazz.	*Zingiber officinale *Rosc.
	(Huang Qin) 15 g	(Zhe Bei Mu) 15 g	(Pu Gong Ying) 15 g	(Gan Jiang) 10 g
	*Trichosanthes kirilowii *Maxim.	*Pinellia ternata *(Thunb) Breit.	*Coptis chinensis *Franch.	*Radix Glycyrrhizae preparata*
	(Quan Gua Lou) 10 g	(Ban Xia) 9 g	(Huang Lian) 6 g	(Zhi Gan Cao) 6 g
	*Santalum album *L.			
	(Tan Xiang) 5 g			
Shou 2015 [30]	*Scutellaria baicalensis *Georgi	*Pinellia ternata *(Thunb) Breit.	*Ziziphus jujuba *Mill	*Pseudostellaria heterophylla *(Miq.) Pax ex pax et Hoffm.
	(Huang Qin) 10 g	(Zhi Ban Xia) 10 g	(Da Zao) 10 g	(Tai Zi Shen) 15 g
	*Zingiber officinale *Rosc.	*Coptis chinensis *Franch.	*Radix Glycyrrhizae preparata*	
	(Gan Jiang) 6 g	(Huang Lian) 5 g	(Zhi Gan Cao) 5 g	
Yang 2015 [31]	*Pinellia ternata *(Thunb) Breit.	*Scutellaria baicalensis *Georgi	*Zingiber officinale *Rosc.	*Codonopsis pilosula *(Franch.) Nannf.
	(Jiang Ban Xia) 15 g	(Huang Qin) 15 g	(Gan Jiang) 3 g	(Dang Shen) 15 g
	*Coptis chinensis *Franch.	*Ziziphus jujuba *Mill	*Bupleurum chinensis *DC.	*Cynanchum otophyllum*
	(Huang Lian) 3 g	(Da Zao) 9 g	(Chai Hu) 10 g	(Bai Shao) 15 g
	*Citrus aurantium *L.	*Bambusa tuldoides *Munro	*Bletilla striata *(Thunb.) Reichb. F.	*Rubus parvifolius *L.
	(Zhi Shi) 10 g	(Zhu Ru) 9 g	(Bai Ji) 6 g	(Mao Mei Gen) 12 g
	*Evodia rutaecarpa *(Juss.) Benth.	*Radix Glycyrrhizae preparata*		
	(Wu Zhu Yu) 3 g	(Zhi Gan Cao) 10 g		
Wang 2013 [32]	*Coptis chinensis *Franch.	*Scutellaria baicalensis *Georgi	*Codonopsis pilosula *(Franch.) Nannf.	*Pinellia ternata *(Thunb) Breit.
	(Huang Lian) 6 g	(Huang Qin) 10 g	(Dang Shen) 10 g	(Fa Ban Xia) 10 g
	*Radix Glycyrrhizae preparata*	*Zingiber officinale *Rosc.	*Perilla frutescens *(L.) Britt.	*Ophiopogon japonicus *(Thunb.)Ker-Gawl.
	(Zhi Gan Cao) 6 g	(Gan Jiang) 10 g	(Zi Su Geng) 10 g	(Mai Dong) 10 g
	*Fritillaria thunbergii *Miq.	*Citrus aurantium *L.		
	(Zhe Bei Mu) 10 g	(Zhi Qiao) 20 g		
Chen 2013 [33]	*Pinellia ternata *(Thunb) Breit.	*Haematitum*	*Codonopsis pilosula *(Franch.) Nannf.	*Sepiella maindroni de Rochebrune*
	(Qing Ban Xia) 12 g	(Dai Zhe Shi) 15 g	(Dang Shen) 15 g	(Hai Piao Xia) 15 g
	*Zingiber officinale *Rosc.	*Scutellaria baicalensis *Georgi	*Ziziphus jujuba *Mill	*Coptis chinensis *Franch.
	(Gan Jiang) 9 g	(Huang Qin) 9 g	(Da Zao) 6 g	(Huang Lian) 6 g
	*Radix Glycyrrhizae preparata*			
	(Zhi Gan Cao) 3 g			
Sun 2013 [34]	*Bletilla striata *(Thunb.) Reichb. F.	*Citrus aurantium *L.	*Codonopsis pilosula *(Franch.) Nannf.	*Curcuma wenyujin *Y. H. Chen et C. Ling
	(Bai Ji) 30 g	(Zhi Qiao) 12 g	(Dang Shen) 15 g	(Yu Jin) 18 g
	*Coptis chinensis *Franch.	*Scutellaria baicalensis *Georgi	*Ziziphus jujuba *Mill	*Bambusa tuldoides *Munro
	(Huang Lian) 10 g	(Huang Qin) 10 g	(Da Zao) 10 g	(Zhu Ru) 9 g
	*Pinellia ternata *(Thunb) Breit.	*Zingiber officinale *Rosc.	*Radix Glycyrrhizae preparata*	*Evodia rutaecarpa *(Juss.) Benth.
	(Qing Ban Xia) 9 g	(Gan Jiang) 9 g	(Zhi Gan Cao) 6 g	(Wu Zhu Yu) 2 g
Cao 2013 [35]	*Pinellia ternata *(Thunb) Breit.	*Scutellaria baicalensis *Georgi	*Zingiber officinale *Rosc.	*Codonopsis pilosula *(Franch.) Nannf.
	(Fa Ban Xia) 12 g	(Chao Huang Qin) 9 g	(Gan Jiang) 9 g	(Dang Shen) 9 g
	*Coptis chinensis *Franch.	*Ziziphus jujuba *Mill	*Radix Glycyrrhizae preparata*	*Inula japonica *Thunb.
	(Huang Lian) 3 g	(Da Zao) 20 g	(Zhi Gan Cao) 9 g	(Xuan Fu Hua) 12 g
	*Haematitum*			
	(Dai Zhe Shi) 15 g			
Zhu 2012 [36]	*Coptis chinensis *Franch.	*Scutellaria baicalensis *Georgi	*Pinellia ternata *(Thunb) Breit.	*Pseudostellaria heterophylla *(Miq.) Pax ex pax et Hoffm.
	(Huang Lian) 10 g	(Huang Qin) 10 g	(Jiang Ban Xia) 10 g	(Tai Zi Shen) 10 g
	*Zingiber officinale *Rose	*Inula japonica *Thunb.	*Haematitum*	*Evodia rutaecarpa *(Juss.) Benth.
	(Sheng Jiang) 10 g	(Xuan Fu Hua) 15 g	(Dai Zhe Shi) 30 g	(Wu Zhu Yu) 3 g
	*Ziziphus jujuba *Mill	*Radix Glycyrrhizae preparata*		
	(Da Zao) 10 g	(Gan Cao) 8 g		
Shen 2012 [37]	*Pinellia ternata *(Thunb) Breit.	*Scutellaria baicalensis *Georgi	*Coptis chinensis *Franch.	*Pseudostellaria heterophylla *(Miq.) Pax ex pax et Hoffm.
	(Fa Ban Xia) 9 g	(Huang Qin) 6–9 g	(Huang Lian) 3–6 g	(Tai Zi Shen) 9–15 g
	*Zingiber officinale *Rosc.	*Radix Glycyrrhizae preparata*	*Ziziphus jujuba *Mill	
	(Gan Jiang) 6 g	(Zhi Gan Cao) 6 g	(Da Zao) 20 g	
Lu 2010 [38]	*Pinellia ternata *(Thunb) Breit.	*Scutellaria baicalensis *Georgi	*Coptis chinensis *Franch.	*Zingiber officinale *Rosc.
	(Ban Xia) 15 g	(Huang Qin) 12 g	(Huang Lian) 5 g	(Gan Jiang) 3 g
	*Codonopsis pilosula *(Franch.) Nannf.	*Inula japonica *Thunb.	*Haematitum*	*Arca subcrenata *Lischke
	(Dang Shen) 15g	(Xuan Fu Hua) 10 g	(Dai Zhe Shi) 10 g	(Duan Wa Leng Zi) 12 g
	*Cleistocactus sepium*	*Salvia miltiorrhiza *Bge.	*Magnolia officinalis *Rehd.et Wils.	*Radix Glycyrrhizae preparata*
	(Wu Zei Gu) 12 g	(Dan Shen) 15 g	(Hou Po) 10 g	(Gan Cao) 5 g
Chen 2009 [39]	*Pinellia ternata *(Thunb) Breit.	*Scutellaria baicalensis *Georgi	*Coptis chinensis *Franch.	*Zingiber officinale *Rosc.
	(Fa Ban Xia)	(Huang Qin)	(Huang Lian)	(Gan Jiang)
	*Panax ginseng *C. A. Mey.	*Ziziphus jujuba *Mill	*Radix Glycyrrhizae preparata*	*Bupleurum chinensis *DC.
	(Ren Shen)	(Da Zao)	(Gan Cao)	(Chai Hu)
	*Evodia rutaecarpa *(Juss.) Benth.	*Citrus aurantium *L.	*Bambusa tuldoides *Munro	*Inula japonica *Thunb.
	(Wu Zhu Yu)	(Zhi Qiao)	(Zhu Ru)	(Xuan Fu Hua)
	*Haematitum*	*Cynanchum otophyllum*	*Bletilla striata *(Thunb.) Reichb. F.	*Sepiella maindroni de Rochebrune*
	(Dai Zhe Shi)	(Bai Shao)	(Bai Ji)	(Hai Piao Xiao)
Huang and Wu 2007 [40]	*Pinellia ternata *(Thunb) Breit.	*Scutellaria baicalensis *Georgi	*Coptis chinensis *Franch.	*Zingiber officinale *Rosc.
	(Ban Xia) 10 g	(Huang Qin) 10 g	(Huang Lian) 3 g	(Gan Jiang) 7 g
	*Codonopsis pilosula *(Franch.) Nannf.	*Radix Glycyrrhizae preparata*	*Arca subcrenata *Lischke	*Inula japonica *Thunb.
	(Dang Shen) 12 g	(Zhi Gan Cao) 6 g	(Wa Leng Zi) 12 g	(Xuan Fu Hua) 9 g
	*Haematitum*			
	(Dai Zhe Shi) 15 g			

**Table 3:** Evaluation of methodological quality of the included studies.

Study ID	Baseline	Randomization	Double blinding	Withdrawal or dropout	Allocation concealment	Follow-up	Side effects	Jadad scores
He et al. 2016	Comparability	Random number table	NR	E: 2 cases C: 4 cases	NR	NR	NR	3
Shou 2015	Comparability	Mention not described	NR	NR	NR	NR	NR	1
Yang et al. 2015	Comparability	Random number table	NR	NR	NR	6-month recurrence (E: 5 cases C: 8 cases)	NR	2
Wang et al. 2013	Comparability	Random number table	NR	NR	NR	1 week	NR	2
Chen 2013	Comparability	Mention not described	NR	NR	NR	NR	NR	1
Sun et al. 2013	Comparability	Random number table	NR	NR	NR	NR	NR	2
Cao 2013	Comparability	Mention not described	NR	NR	NR	NR	NR	1
Zhu et al. 2012	Comparability	Mention not described	Single-blind	C: 2 cases	NR	NR	8 cases	2
Shen 2012	Comparability	Random number table	NR	NR	NR	NR	NR	2
Lu et al. 2010	Comparability	Mention not described	NR	NR	NR	NR	no	1
Chen et al. 2009	Comparability	Mention not described	NR	NR	NR	12-week recurrence (E: 3 cases C: 11 cases)	C: 14 cases	1
Huang et al. 2007	Comparability	Mention not described	NR	NR	NR	3 months	NR	1

NR: not reported; E: experiment group; C: control group.

## Additional Points

*Supporting Information*. S1 PRISMA Checklist (DOC).

## References

[1] Jain D., Singhal S. (2016). Transoral incisionless fundoplication for refractory gastroesophageal reflux disease: where do we stand?. *Clinical Endoscopy*.

[2] Vakil N., van Zanten S. V., Kahrilas P. (2006). The Montreal definition and classification of gastroesophageal reflux disease: a global evidence-based consensus. *The American Journal of Gastroenterology*.

[3] Jung H.-K. (2011). Epidemiology of gastroesophageal reflux disease in asia: a systematic review. *Journal of Neurogastroenterology and Motility*.

[4] Nocon M., Labenz J., Jaspersen D. (2009). Health-related quality of life in patients with gastro-oesophageal reflux disease under routine care: 5-year follow-up results of the ProGERD study. *Alimentary Pharmacology and Therapeutics*.

[5] Wahlqvist P., Karlsson M., Johnson D., Carlsson J., Bolge S. C., Wallander M.-A. (2008). Relationship between symptom load of gastro-oesophageal reflux disease and health-related quality of life, work productivity, resource utilization and concomitant diseases: survey of a US cohort. *Alimentary Pharmacology and Therapeutics*.

[6] Vakil N. (2008). New pharmacological agents for the treatment of gastroesophageal reflux disease. *Reviews in Gastroenterological Disorders*.

[7] Goh K. L., Choi M. G., Hsu P. I. (2016). Pharmacological and safety profile of dexlansoprazole: a new proton pump inhibitor—implications for treatment of gastroesophageal reflux disease in the Asia pacific region. *Journal of Neurogastroenterology and Motility*.

[8] Ngamruengphong S., Leontiadis G. I., Radhi S., Dentino A., Nugent K. (2011). Proton pump inhibitors and risk of fracture: a systematic review and meta-analysis of observational studies. *American Journal of Gastroenterology*.

[9] Eom C., Park S. M., Myung S., Yun J. M., Ahn J. (2011). Use of acid-suppressive drugs and risk of fracture: a meta-analysis of observational studies. *The Annals of Family Medicine*.

[10] Ye X., Liu H., Wu C. (2011). Proton pump inhibitors therapy and risk of hip fracture. *European Journal of Gastroenterology & Hepatology*.

[11] Sultan N., Nazareno J., Gregor J. (2008). Association between proton pump inhibitors and respiratory infections: a systematic review and meta-analysis of clinical trials. *Canadian Journal of Gastroenterology*.

[12] Lambert A. A., Lam J. O., Paik J. J., Ugarte-Gil C., Drummond M. B., Crowell T. A. (2015). Risk of community-acquired pneumonia with outpatient proton-pump inhibitor therapy: a systematic review and meta-analysis. *PLoS ONE*.

[13] Giuliano C., Wilhelm S. M., Kale-Pradhan P. B. (2012). Are proton pump inhibitors associated with the development of community-acquired pneumonia? A meta-analysis. *Expert Review of Clinical Pharmacology*.

[14] Trikudanathan G., Israel J., Cappa J., O'Sullivan D. M. (2011). Association between proton pump inhibitors and spontaneous bacterial peritonitis in cirrhotic patients—a systematic review and meta-analysis. *International Journal of Clinical Practice*.

[15] Deshpande A., Pant C., Pasupuleti V. (2012). Association between proton pump inhibitor therapy and clostridium difficile infection in a meta-analysis. *Clinical Gastroenterology and Hepatology*.

[16] Kwok C. S., Arthur A. K., Anibueze C. I., Singh S., Cavallazzi R., Loke Y. K. (2012). Risk of clostridium difficile infection with acid suppressing drugs and antibiotics: meta-analysis. *American Journal of Gastroenterology*.

[17] Janarthanan S., Ditah I., Adler D. G., Ehrinpreis M. N. (2012). Clostridium difficile-associated diarrhea and proton pump inhibitor therapy: a meta-analysis. *The American Journal of Gastroenterology*.

[18] Li Y. J., Wang Y. L., Wei W. (2015). Systematic system evaluation on randomized controlled trials and meta analysis on gastroesophageal reflux disease treated with Xinkai Kujiang method. *World Journal of Integrated Traditional and Western Medicine*.

[19] Zhao Y.-H., Liu Z.-L., Li L.-H., Jiang S.-H., Shi C.-H. (2012). Systematic review of randomized controlled trials of traditional Chinese medicine treatment of non-acute bronchial asthma complicated by gastroesophageal reflux. *Journal of Traditional Chinese Medicine*.

[20] Xu G. (2006). Treatment of reflux laryngopharyngitis with modified Banxia Xiexin Tang (Pinellia Decoction for Draining the Heart)—a report of 40 cases. *Journal of Traditional Chinese Medicine*.

[21] Huedo-Medina T. B., Sánchez-Meca J., Marín-Martínez F., Botella J. (2006). Assessing heterogeneity in meta-analysis: Q statistic or I^2^ Index?. *Psychological Methods*.

[22] Zheng X. Y. (2002). *Chinese Herbal Medicine New Medicine Clinical Research Guiding Principle*.

[23] Devault K. R., Castell D. O. (1999). Updated guidelines for the diagnosis and treatment of gastroesophageal reflux disease. *American Journal of Gastroenterology*.

[24] Chinese Society of Digestive Endoscopy (2003). Diagnosis and treatment guidelines on relux esophagitis. *Chinese Journal of Digestive Endoscopy*.

[25] Savović J., Weeks L., Sterne J. A. C. (2014). Evaluation of the Cochrane Collaboration's tool for assessing the risk of bias in randomized trials: focus groups, online survey, proposed recommendations and their implementation. *Systematic Reviews*.

[26] Jadad A. R., Moore R. A., Carroll D. (1996). Assessing the quality of reports of randomized clinical trials: is blinding necessary?. *Controlled Clinical Trials*.

[27] Higgins J. P. T., Thompson S. G., Deeks J. J., Altman D. G. (2003). Measuring inconsistency in meta-analyses. *British Medical Journal*.

[28] DerSimonian R., Laird N. (1986). Meta-analysis in clinical trials. *Controlled Clinical Trials*.

[29] He H., Han X. F. (2016). Clinical observation of the Banxia Xiexin Decoction on treating non-erosive reflux disease with stagnated heat in liver and stomach syndrome. *Zhejiang Journal of Traditional Chinese Medicine*.

[30] Shou H. Q. (2015). Efficacy of the Banxia Xiexin decoction on gastroesophageal reflux disease. *Clinical Journal of Chinese Medicine*.

[31] Yang Y. Y., Chen Y. Q., Wu T. T. (2015). Combination of ‘Banxia Xiexin Decoction’ and ‘Sini Powder’ for the treatment of reflux esophagitis. *Shanghai Journal of Traditional Chinese Medicine*.

[32] Wang J. H., Zhang Y. M., Wang C. W., Hao S. Y. (2013). Effect of modified Banxia Xiexin Decoction cooperated with point injection on treating 56 cases of gastroesophageal reflux disease (GERD). *Journal of Sichuan of Traditional Chinese Medicine*.

[33] Chen X. (2013). Clinical curative effect of the Banxia Xiexin decoction on treating 58 cases of GERD. *Clinical Journal of Chinese Medicine*.

[34] Sun X. H., Niu R., Yang J. Q. (2013). Clinical observation of Xinkai Kujiang therapy on treating 60 cases of gastroesophageal reflux disease. *Global Traditional Chinese Medicine*.

[35] Cao W. Y. (2013). Modified Banxia Xiexin decoction on treating 32 case of reflux esophagitis. *Chinese Journal of Integrated Traditional and Western Medicine*.

[36] Zhu Z. Z., Hao J. J., Lv J., Wang L. L., Zhu J. J. (2012). Clinical observation of Banxia Xiexin decoction plus Xuanfu Daizhe decoction on treating 120 cases of reflux esophagitis. *Research of Integrated Traditional Chinese and Western Medicine*.

[37] Shen Y. L. (Aug 2012). Banxia Xiexin Tang in treating reflux esophagitis curative effect observation. *Journal of Liaoning University of TCM*.

[38] Lu W., Liu X. W., Li Y. (2010). Banxia Xiexin decoction on treating reflux esophagitis. *Chinese Medicine Modern Distance Education of China*.

[39] Chen K. Y., Liu H., Zeng Y. P., Luo J. H. (2009). Clinical observation of modified Banxia Xiexin decoction on treating 90 cases of reflux esophagitis. *Journal of New Chinese Medicine*.

[40] Huang D. H., Wu Y. N. (2007). Clinical observation of Banxia Xiexin decoction plus-minus in the treatment of 60 patients with reflux esophagitis. *China Medical Herald*.

[41] Dunbar K. B., Agoston A. T., Odze R. D. (2016). Association of acute gastroesophageal reflux disease with esophageal histologic changes. *The Journal of the American Medical Association*.

[42] Xie C., Wang J., Li Y. (2017). Esophagogastric junction contractility integral reflect the anti-reflux barrier dysfunction in patients with gastroesophageal reflux disease. *Journal of Neurogastroenterology and Motility*.

[43] Nadaleto B. F., Herbella F. A. M., Patti M. G. (2016). Gastroesophageal reflux disease in the obese: pathophysiology and treatment. *Surgery*.

[44] Wright C. E., Ebrecht M., Mitchell R., Anggiansah A., Weinman J. (2005). The effect of psychological stress on symptom severity and perception in patients with gastro-oesophageal reflux. *Journal of Psychosomatic Research*.

[45] Baker L. H., Lieberman D., Oehlke M. (1995). Psychological distress in patients with gastroesophageal reflux disease. *The American Journal of Gastroenterology*.

[46] Dore M. P., Pes G. M., Bassotti G., Farina M. A., Marras G., Graham D. Y. (2016). Risk factors for erosive and non-erosive gastroesophageal reflux disease and Barrett's esophagus in Nothern Sardinia. *Scandinavian Journal of Gastroenterology*.

[47] Banovcin P., Halicka J., Halickova M. (2016). Studies on the regulation of transient lower esophageal sphincter relaxations (TLESRs) by acid in the esophagus and stomach. *Diseases of the Esophagus*.

[48] Pan S., Lan Q. Q., Lin S. N., Zhu Y. P., Xu Z. F. (2012). Research of pinelliae decoction for purging stomach-fire on immune function of Esophagus Mucous membrane in rat model of reflux esophagitis. *Shanxi Journal of Traditional Chinese Medicine*.

[49] Tang Y.-P., Liu S.-M., Wei W. (2014). Effect of pungent dispersion bitter purgation method on the esophageal mucosal intercellular space of reflux esophagitis model rats. *Chinese Journal of Integrated Traditional and Western Medicine*.

[50] Liu X. N., Jin X. D., Li Y. Z., Liu G. L., Sun W. (2008). Influence of BX decoction on mRNA expression for calponin and caldesmon and intracellular free calcium. *Chinese Journal of Experimental Traditional Medical Formulae*.

[51] Liu X. N., Jin X. D., Li Y. Z., Liu G. L., Sun W. (2008). Study on the treatment effect of BX decoction on rat models of reflux esophagitis. *Journal of Radioimmunology*.

